# Central nervous system involvement and relapse in adult acute lymphoblastic leukaemia—a population‐based analysis in 2007–2022

**DOI:** 10.1111/bjh.70589

**Published:** 2026-06-01

**Authors:** Erik Boberg, Anna Lübking, Emma Bergfelt Lennmyr, Caroline E. Dietrich, Joel Joelsson, Karin E. Smedby, Tove Wästerlid, Helene Hallböök

**Affiliations:** ^1^ Division of Clinical Epidemiology, Department of Medicine Solna Karolinska Institutet Stockholm Sweden; ^2^ Department of Haematology Karolinska University Hospital Stockholm Sweden; ^3^ Department of Haematology Oncology and Radiation Physics, Skåne University Hospital Lund Sweden; ^4^ Department of Medical Sciences Uppsala University Uppsala Sweden; ^5^ Department of Medicine Uppsala University Hospital Uppsala Sweden

**Keywords:** acute lymphoblastic Leukaemia, adult, central nervous system, population‐based, relapse

## Abstract

Central nervous system (CNS) involvement and relapse remains a clinical challenge in the management of acute lymphoblastic leukaemia (ALL), and studies among adult ALL patients are scarce. We identified all adult ALL patients in Sweden between 2007 and 2022 (*n* = 838) from the population‐based ALL register and evaluated factors associated with CNS involvement and relapse. CNS involvement at diagnosis was detected in 7.8% of patients, more often in patients with T‐cell ALL (T‐ALL) and with white blood cell (WBC) count >30 × 10^9^/L, without affecting overall survival or risk of any relapse. The 5‐year cumulative incidence of CNS relapse was 4.6% (95% confidence interval [CI] 3.1–6.6), with WBC count >100 × 10^9^/L and male sex as independent risk factors. Furthermore, CNS involvement at diagnosis increased the rate of CNS relapse in B‐cell ALL (B‐ALL) (hazard ratio [HR] 4.52, 95% CI 1.35–15.2), but not T‐ALL (HR 0.94, 95% CI 0.12–7.53). Neither Philadelphia chromosome status nor *KMT2A::AFF1* was associated with CNS involvement at diagnosis or CNS relapse. Patients with CNS relapse had a 2‐year overall survival of 34% (13% if relapse occurred <12 months from diagnosis). We conclude that contemporary treatment protocols can prevent CNS relapse for most patients but suggest that B‐ALL patients with CNS involvement may have an unmet need for improved CNS‐directed therapy.

## INTRODUCTION

Without central nervous system (CNS)‐directed treatment, 30%–40% of acute lymphoblastic leukaemia (ALL) patients will experience a CNS relapse,^1^ most likely due to survival of leukaemic blasts present in the CNS already at diagnosis.^2^ Minimizing the risk for CNS relapse is of vast importance as it is associated with a median survival of less than 1 year among adult ALL patients.^3^, ^4^, ^5^ Therefore, contemporary ALL protocols include CNS‐directed intravenous and intrathecal chemotherapy,^6^ reducing the CNS relapse risk to about 7%.^4^, ^5^, ^7^ However, such chemotherapy can cause both acute toxicity and long‐term neurocognitive impairments.^8^, ^9^ Methotrexate (MTX) is regarded as the major culprit, associated with the risk of leucoencephalopathy and both short‐ and long‐term neurotoxicity.^10^, ^11^

Conventional cerebrospinal fluid (CSF) cytology remains the gold standard for diagnosing CNS involvement, and detection of leukaemic blasts in the CSF at diagnosis is considered a high‐risk feature in some adult ALL protocols, leading to reinforced CNS‐directed therapy. However, the impact of CNS involvement on subsequent CNS relapse risk varies across studies^5^, ^12^, ^13^, ^14^, ^15^ and several reports suggest that CSF cytology lacks sensitivity, specificity and reproducibility.^16^, ^17^

Commonly reported factors associated with CNS involvement at diagnosis and CNS relapse include high white blood cell (WBC) count, T‐ALL phenotype and high‐risk genetics such as presence of the translocations *BCR::ABL1* or *KMT2A::AFF1*.^12^, ^13^, ^14^, ^18^ The data supporting these claims predominantly come from clinical trials, which often include highly selected patients with outcomes driven by the treatment protocol studied, hampering external validity. Additionally, there are few studies on adult ALL cohorts, especially including older adults,^4^, ^19^ compared to paediatric ALL.

Improved understanding of which patients who need more extensive CNS prophylaxis can potentially reduce both excessive toxicity from overtreatment and relapses due to undertreatment. Therefore, this study aimed to quantify potential risk factors and outcomes associated with CNS involvement at diagnosis and relapse, using a consecutive, national cohort of adult patients diagnosed with ALL in Sweden between 2007 and 2022, with virtually complete coverage.

## MATERIALS AND METHODS

### Data sources

This cohort study included all patients diagnosed with ALL at a Swedish adult haematology clinic and thus registered in the Swedish ALL register between 2007 and 2022. The Swedish ALL register has a coverage of 98%^20^ of adult ALL patients compared to the legally mandated Swedish Cancer Register and contains detailed diagnostic and clinical data.

Recently, a validation study was conducted on the Swedish ALL register,^21^ in which existing register data for 139 ALL patients aged 46–65, diagnosed 2013–2021 was compared with the corresponding source medical records data. For the variable ‘CNS disease at diagnosis’, the exact agreement was 93.9% with a Cohen's kappa of 0.76, while ‘CNS relapse’ had an exact agreement of 89.3% and Cohen's kappa of 0.34. Missing information on these variables was reported for one and no patients respectively. The paradoxically low Cohen's kappa for CNS relapses despite a high exact agreement can be explained in part by a low prevalence of CNS relapses in the validation cohort. Furthermore, for the purposes of the present study, we re‐evaluated medical records of two patients registered with CNS relapse and found that they had been incorrectly recorded as negative during validation, underestimating the true Cohen's kappa.

Vital status at the end of study was obtained from the Swedish Population Register. This study included only T‐cell ALL (T‐ALL) and B‐cell ALL (B‐ALL), thus excluding patients with Burkitt leukaemia (*n* = 39), B‐lymphoblastic lymphoma (*n* = 1) and ALL not otherwise specified (*n* = 44). The final study population comprised 838 patients diagnosed with ALL, aged 17–94 years.

### Subpopulations

Based on the underlying study population, the following analysis‐specific populations were constructed (Figure S1): The analysis of factors associated with CNS involvement at diagnosis excluded patients without known CNS status at diagnosis. Analyses of outcomes in patients with and without CNS involvement at diagnosis, risk factors for CNS relapse and outcomes after CNS relapse excluded patients who were primarily treated with palliative intent. Additionally, patients older than 70 years at diagnosis were excluded from these analyses, as older patients are often not evaluated for CNS involvement at relapse.

### Definitions

CNS involvement at diagnosis or relapse was defined as either (a) at least five leukaemic blasts per microlitre in the CSF by conventional cytology (equal to CNS3 status in the Nordic Society of Paediatric Haematology and Oncology ALL2008 protocol),^22^ (b) presence of leukaemic infiltrates on computer tomography or magnetic resonance imaging, (c) cranial nerve palsies or (d) presence of intraocular leukaemia on fundoscopy. Cut‐off values for WBC counts were defined as <30, 30–100 and >100 × 10^9^/L, based on previous studies.^5^, ^12^, ^14^ Bulky disease was defined as a mediastinal mass greater than one‐third of the thoracic width or >10 cm in diameter. Early CNS relapses were defined as a CNS relapse occurring within 12 months from initial diagnosis in accordance with previous studies.^4^, ^5^, ^7^, ^23^ Age groups (17–45, 46–60, 61–70, 71+) were constructed to align with age cut‐offs for ALL treatments in the Swedish national guidelines.

### Statistical analysis

Odds ratios (ORs) with 95% confidence intervals (CIs) were estimated using univariable and multivariable logistic regression to quantify associations between clinical factors and CNS involvement at diagnosis. A set of adjustments was used, based on a priori selection of potential confounders. The association between CNS involvement at diagnosis and complete remission was analysed using a chi‐square test.

To analyse the impact of CNS involvement at diagnosis on prognosis, methods in survival analysis were used.

Patients were followed from diagnosis until death, 10 years from ALL diagnosis or end of study (31 December 2022). Overall survival (OS) was estimated using the Kaplan–Meier method. Log rank tests were used to test for differences in OS. Cause‐specific cumulative incidence functions (CIFs) for relapse and CNS relapse were calculated non‐parametrically using the Aalen–Johansen estimator, with death from any cause and non‐CNS relapse as competing risks. Grey's test was used to test for differences in CIF.

Adjusted hazard ratios (HRs) with 95% CIs comparing all‐cause mortality and relapse rates were estimated using Cox proportional hazards models. The proportional hazards assumption was formally assessed using the Schoenfeld test of residuals. The assumption of proportional hazards was fulfilled for all variables except CNS involvement (Schoenfeld residual *p* = 0.03). Graphical evaluation of the non‐parametric hazard rates by CNS involvement showed a slight increased relative relapse rate over time with no clinical explanation. Therefore, the proportional hazards assumption remained throughout all models. Age was modelled as a cubic spline with 2 df to allow for a non‐linear effect.

As a sensitivity analysis, the effect of WBC count at diagnosis on^1^ CNS involvement at diagnosis and^2^ CNS relapse was investigated. For CNS involvement, a logistic regression model was fitted, and for CNS relapse, a Cox proportional hazards model was used. For both outcomes, the effect of WBC count was allowed to be non‐linear using cubic splines with 2 df.

All tests were two‐sided, with *p* < 0.05 denoting a statistically significant difference, and CIs were calculated at the 95% level. All models were complete case.

All statistical analyses were performed using the survival, tidycmprsk and R splines packages in R version 4.4.2.^24^ The study was approved by the national Swedish Ethical Review Authority (number 2022‐05354‐01, 2023‐04944‐02). All participants consented to inclusion in the Swedish ALL Register.

## RESULTS

### Patient and disease characteristics and treatment

Characteristics of the 838 patients diagnosed with ALL between 2007 and 2022 are presented in Table 1. Median follow‐up was 2.2 years (interquartile range [IQR] 0.9–6.35) for all patients and 5.7 years (IQR 2.64–9.98) for patients alive at the end of the study. Seventeen per cent of patients had T‐ALL and 83% had B‐ALL. Genetic analysis was performed in 92% of patients. *BCR::ABL1* was present in 30% (36% of B‐ALL and 2% of T‐ALL) and *KMT2A::AFF1* in 3.9% (4% of B‐ALL and 2% of T‐ALL).

**TABLE 1:** Patient and disease characteristics of the included adult ALL patients diagnosed between 2007 and 2022.

Characteristics	*n* (col %)
Total	838 (100)
Age	
17–45	349 (42)
46–60	155 (18)
61–70	147 (18)
71+	187 (22)
Median (range)	52 (17–94)
Sex	
F	364 (43)
M	474 (57)
WHO Performance Status	
0–1	681 (81)
2–4	136 (16)
Unknown	21 (2.5)
ALL type	
B‐ALL	692 (83)
T‐ALL	146 (17)
Genetic analysis performed	
Yes	773 (92)
*BCR::ABL1*	236 (30)
*KMT2A::AFF1*	30 (3.9)
No	65 (8)
WBC at diagnosis	
WBC <30	560 (67)
WBC 30–100	159 (19)
WBC >100	119 (14)
*Median (range)*	12 (0–982)
Bulky disease at diagnosis	
No	713 (85)
Yes	42 (5.0)
Unknown	83 (9.9)
CNS involvement at diagnosis	
CNS–	691 (82)
CNS+	59 (7.0)
Unknown	88 (11)
Treatment protocol	
Paediatric/paediatric inspired	318 (38)
ABCDV protocol	191 (23)
EWALL inspired	117 (14)
Palliative/no treatment	89 (11)
Other	91 (11)
Unknown	32 (3.8)
aHSCT in primary treatment	
Yes	207 (25)
No	93 (11)
Unknown	538 (64)

Abbreviations: ABCDV, Cytarabine, Betamethasone, Cyclophosphamide, Daunorubicin and Vincristine; aHSCT, allogeneic haematopoietic stem cell transplantation; ALL, acute lymphoblastic leukaemia; CNS, central nervous system; EWALL, European Working Group for Adult ALL; WBC, white blood cells.

The choice of treatment generally followed the national Swedish guidelines including systemic high‐dose intravenous MTX and/or cytarabine (ARA‐C) and intrathecal MTX (with or without addition of ARA‐C and prednisone) for CNS‐directed therapy. Only high‐risk patients who were consolidated with allogeneic haematopoietic stem cell transplant (aHSCT) in first complete remission (CR1) received cranial or craniospinal irradiation (Figure S2). CNS involvement at diagnosis per se was generally not considered an indication for aHSCT and consolidation with aHSCT in CR1 was performed in 11 patients (19%) with CNS involvement and 188 patients (27%) without CNS involvement (data not shown).

### CNS involvement at diagnosis

CNS involvement at diagnosis was assessed in 750 patients (89%) among whom 59 (7.9%) had such involvement (Table 2). After adjustment for confounders, age 61–70 at diagnosis (OR 2.23, 95% CI 1.13–4.36), T‐ALL phenotype (OR 2.15, 95% CI 1.15–3.90) and high WBC count >100 × 10^9^/L (OR 2.55, 95% CI 1.24–5.09) were found significantly associated with CNS involvement (Table 2). Since *BCR::ABL1* is almost exclusively present in B‐ALL, and bulky disease is almost exclusively present in T‐ALL, these factors were evaluated in separate models stratified by ALL type (Table S1). Neither factor was associated with CNS involvement at diagnosis.

**TABLE 2:** Clinical characteristics of patients with and without CNS involvement at diagnosis as well as their associations to CNS involvement using unadjusted and adjusted odds ratios (ORs) with 95% confidence intervals (CIs).

Characteristic	Data		Univariable	Multivariable
	CNS–	CNS+	OR (95% CI)^1^	OR (95% CI)^2^
	*N* = 691 (92%)	*n* = 59 (7.9%)		
Age, *n* (row %)				
17–45	313 (93)	23 (6.8)	1.00 (ref)	1.00 (ref)
46–60	134 (92)	11 (7.6)	1.12 (0.51–2.31)	1.15 (0.52–2.38)
61–70	115 (87)	17 (13)	2.01 (1.02–3.89)	2.23 (1.13–4.36)
71+	129 (94)	8 (5.8)	0.84 (0.35–1.86)	0.89 (0.37–1.98)
Sex, *n* (row %)				
F	302 (94)	19 (5.9)	1.00 (ref)	1.00 (ref)
M	389 (91)	40 (9.3)	1.63 (0.94–2.94)	1.78 (1.01–3.22)
				OR (95% CI)^3^ (*n* = 750)
ALL type, *n* (row %)				
B‐ALL	572 (93)	41 (6.7)	1.00 (ref)	1.00 (ref)
T‐ALL	119 (87)	18 (13)	2.11 (1.15–3.75)	2.15 (1.15–3.90)
				OR (95% CI)^4^ (*n* = 705)
WBC at diagnosis, *n* (row %)				
WBC <30	477 (95)	27 (5.4)	1.00 (ref)	1.00 (ref)
WBC 30–100	120 (88)	16 (12)	2.36 (1.21–4.47)	2.02 (0.99–3.94)
WBC >100	94 (85)	16 (15)	3.01 (1.53–5.74)	2.55 (1.24–5.09)
*KMT2A::AFF1*, *n* (row %)				
No	623 (92)	54 (8.0)	1.00 (ref)	1.00 (ref)
Yes	25 (89)	3 (11)	1.38 (0.32–4.11)	1.03 (0.23–3.32)
Unknown	43 (96)	2 (4.4)		
				OR (95% CI)^5^ (*n* = 587)
*BCR::ABL1* (B‐ALL only), *n* (row %)				
No	355 (93)	25 (6.6)	1.00 (ref)	1.00 (ref)
Yes	192 (92)	16 (7.7)	1.18 (0.61–2.25)	0.90 (0.41–1.89)
Unknown	25 (100)	0 (0)		
				OR (95% CI)^6^ (*n* = 125)
Bulky disease at diagnosis (T‐ALL only), *n* (row %)				
No	75 (84)	14 (16)	1.00 (ref)	1.00 (ref)
Yes	33 (92)	3 (8.3)	0.49 (0.11–1.62)	0.62 (0.13–2.24)
Unknown	11 (92)	1 (8.3)		

Abbreviations: ALL, acute lymphoblastic leukaemia; CI, confidence interval; CNS, central nervous system; OR, odds ratio; WBC, white blood cell.

^1^ Estimated from univariable logistic regression models.

^2^ Estimated from a multivariable logistic regression model including age and sex.

^3^ Estimated from a multivariable logistic regression model including age, sex and ALL type.

^4^ Estimated from a multivariable logistic regression model including age, sex, ALL type, WBC at diagnosis and *KMT2A::AFF1*.

^5^ B‐ALL patients only. Estimated from a multivariable logistic regression model including age, sex, WBC count, *KMT2A::AFF1* and *BCR::ABL1*.

^6^ T‐ALL patients only. Estimated from a multivariable logistic regression model including age, sex, WBC count and bulky disease at diagnosis.

^3–6^In the multivariable models 3–6, age was modelled as a cubic spline with 2 df.

**FIGURE 1:**
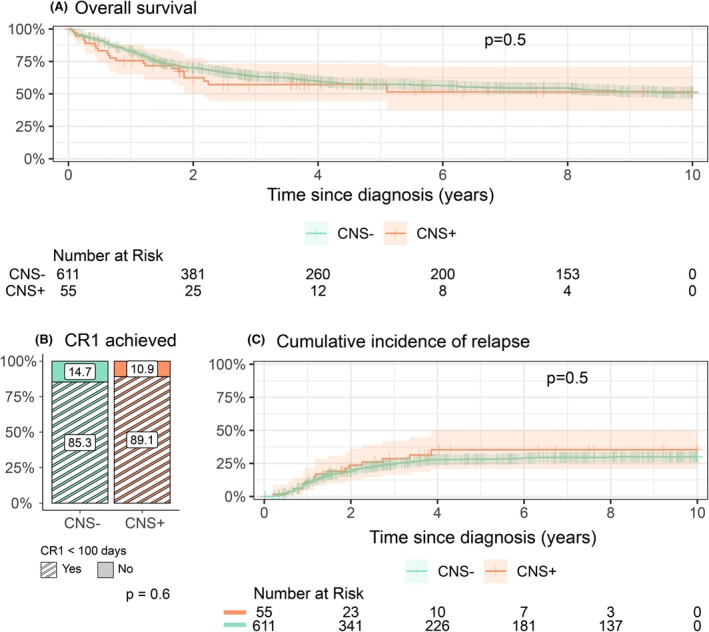
Outcomes in patients with and without central nervous system (CNS) involvement at diagnosis. (A) Overall survival. *p*‐value from log rank test. (B) Proportion of patients reaching first complete remission (CR1) within 100 days from diagnosis. (C) Cause‐specific cumulative risk of relapse at any site, accounting for competing risks. *p*‐value from Grey's test.

Next, we compared treatment outcomes among patients with and without CNS involvement at diagnosis (Figure 1). Complete remission was reached in a similar proportion of patients in both groups within 100 days from diagnosis (89.1% vs. 85.3%, *p* = 0.6 from chi‐square test). Moreover, no differences in OS or risk of relapse were observed (*p* = 0.5 from both log‐rank and Grey's test). After adjusting for potential confounders, including age, these results remained (Table S2).

### CNS relapse

**FIGURE 2:**
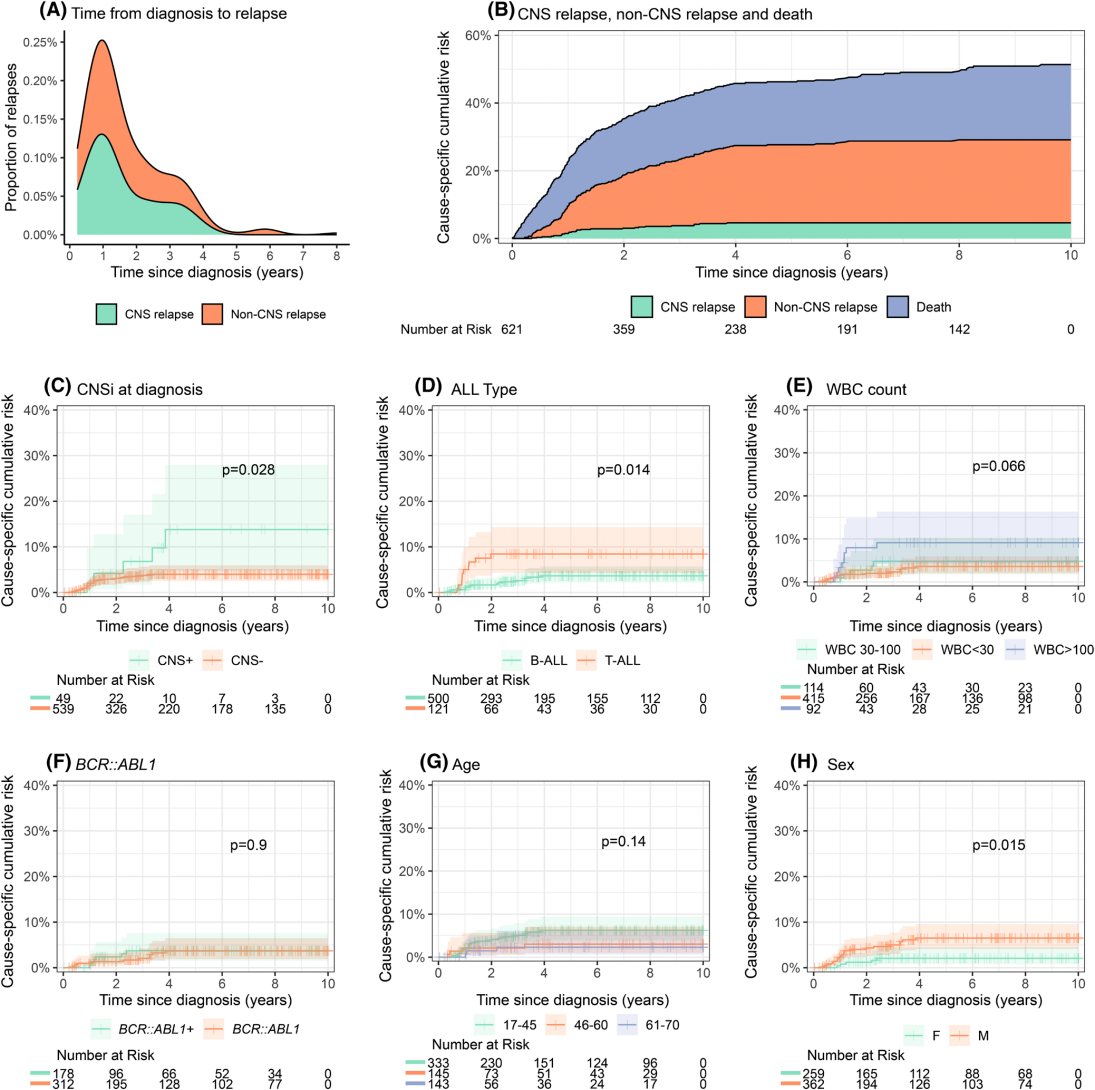
Central nervous system (CNS) relapse over time since diagnosis. (A) Density plot of time from diagnosis to relapse. (B) Cumulative risk of CNS relapse (5‐year point estimates: 4.6%, 95% confidence interval [CI] 3.1–6.6), non‐CNS relapse and death from any cause. (C–G) Cumulative risk of CNS relapse by CNS involvement at diagnosis, acute lymphoblastic leukaemia (ALL) type, white blood cell (WBC) count, *BCR::ABL1* status, age and sex. The analysis includes patients 70 years of age or younger and excludes patients who only received palliative treatment (*n* = 621). *p*‐values from Grey's test.

Of the 621 patients who were 70 years old or younger at diagnosis and did not receive treatment with palliative intent, 26 patients experienced a first relapse involving the CNS (16% of all first relapses). Most CNS relapses occurred within 5 years from diagnosis and the distribution of CNS relapses over time was similar to non‐CNS relapses (Figure 2A). The 5‐year cumulative incidence of CNS relapse was 4.6% (95% CI 3.1%–6.6%), in the presence of the competing risks of death and non‐CNS relapse (Figure 2B). The characteristics of the 26 patients with CNS relapse are provided in Table 3. The median age at relapse was 33 years (range 19–69) and 81% were male. Five patients (19%) had CNS involvement at initial diagnosis. Relapse was confined to the CNS in 19 patients (73%), while 27% had combined CNS with bone marrow or other involvements. Notably, while 23% of patients were *BCR::ABL1*‐positive, none had *KMT2A::AFF1*. Four patients (15%) had previously undergone aHSCT. Reinduction was given to 24 patients (92%) and 10 patients underwent aHSCT within 180 days after the relapse.

**TABLE 3:** Characteristics of adult patients with CNS relapse after treatment for acute lymphoblastic leukaemia that were 70 years old or younger at diagnosis and were not treated with palliative intent.

Characteristics	*n* (col %)
Total	26 (100)
Age at CNS relapse, median (range)	33 (19–69)
Sex	
F	5 (19)
M	21 (81)
ALL type	
B‐ALL	16 (62)
T‐ALL	10 (38)
*BCR::ABL1*	
No	20 (77)
Yes	6 (23)
*KMT2A::AFF1*	
No	26 (100)
Yes	0 (0)
CNS involvement at diagnosis	
CNS–	20 (77)
CNS+	5 (19)
Unknown	1 (3.8)
WBC at diagnosis	
WBC <30	13 (50)
WBC 30–100	5 (19)
WBC >100	8 (31)
Time from diagnosis to relapse	
≤1 year	9 (35)
>1 year	17 (65)
Relapse sites	
CNS + BM	6 (23)
CNS + Other	1 (3.8)
Isolated CNS	19 (73)
Relapse after aHSCT	
Yes	4 (15)
No	22 (85)
Treatment	
No reinduction	2 (8)
Reinduction, but no aHSCT ≤180 days after relapse	14 (54)
aHSCT ≤180 days after relapse	10 (38)

Abbreviations: aHSCT, allogeneic haematopoietic stem cell transplantation; ALL, acute lymphoblastic leukaemia; CNS, central nervous system; CR2, second complete remission; WBC, white blood cells.

**FIGURE 3:**
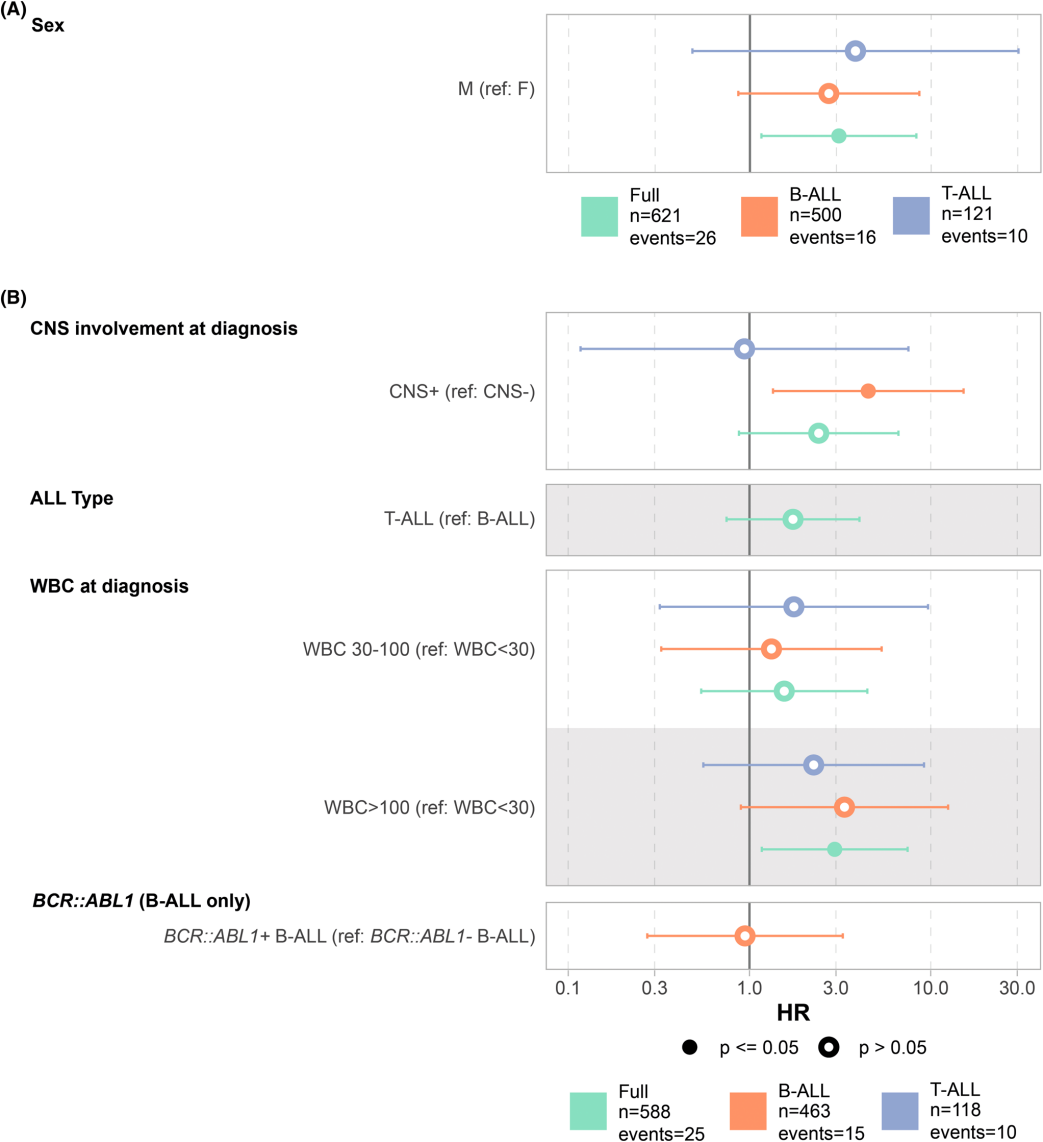
Hazard ratios (HRs) with 95% confidence intervals comparing relative rates of central nervous system (CNS) relapse in patients aged up to 70 years at diagnosis excluding patients primarily treated with palliative intent. (A) Estimated from cause‐specific Cox proportional hazards models including age and sex. (B) Estimated from cause‐specific Cox proportional hazards models. The full model included age, sex, CNS involvement at diagnosis, acute lymphoblastic leukaemia (ALL) type and white blood cell (WBC) count at diagnosis. The B‐ALL model included age, sex, CNS involvement at diagnosis, WBC count at diagnosis and *BCR::ABL1* status. The T‐ALL model included age, sex, CNS involvement at diagnosis and WBC count at diagnosis. Age was modelled as a natural cubic spline with 2 knots in all models.

Next, we evaluated how CNS involvement and other factors at diagnosis influenced the risk for CNS relapse (Figure 2C–H). The cumulative incidence of CNS relapse was significantly higher in males, those with CNS involvement at diagnosis, and in T‐ALL patients. Based on adjusted analyses, male sex (HR 3.10, 95% CI 1.16–8.29) and WBC count >100 (HR 2.95, 95% CI 1.17–7.45) at diagnosis were independent risk factors (Figure 3). When stratifying by ALL type, CNS involvement at diagnosis predicted the rate of CNS relapse in B‐ALL patients (HR 4.52, 95% CI 1.35–15.2), but not in T‐ALL patients (HR 0.94, 95% CI 0.12–7.53, Figure 3).

To further elucidate the observed association between WBC count with both CNS involvement at diagnosis and CNS relapse rate, unadjusted models were fitted with WBC count modelled as a cubic spline. At WBC levels <200 (which most patients had, Figure S3A), there was an almost linear relationship to OR of CNS involvement and HR for CNS relapse (Figure S3B,C).

**FIGURE 4:**
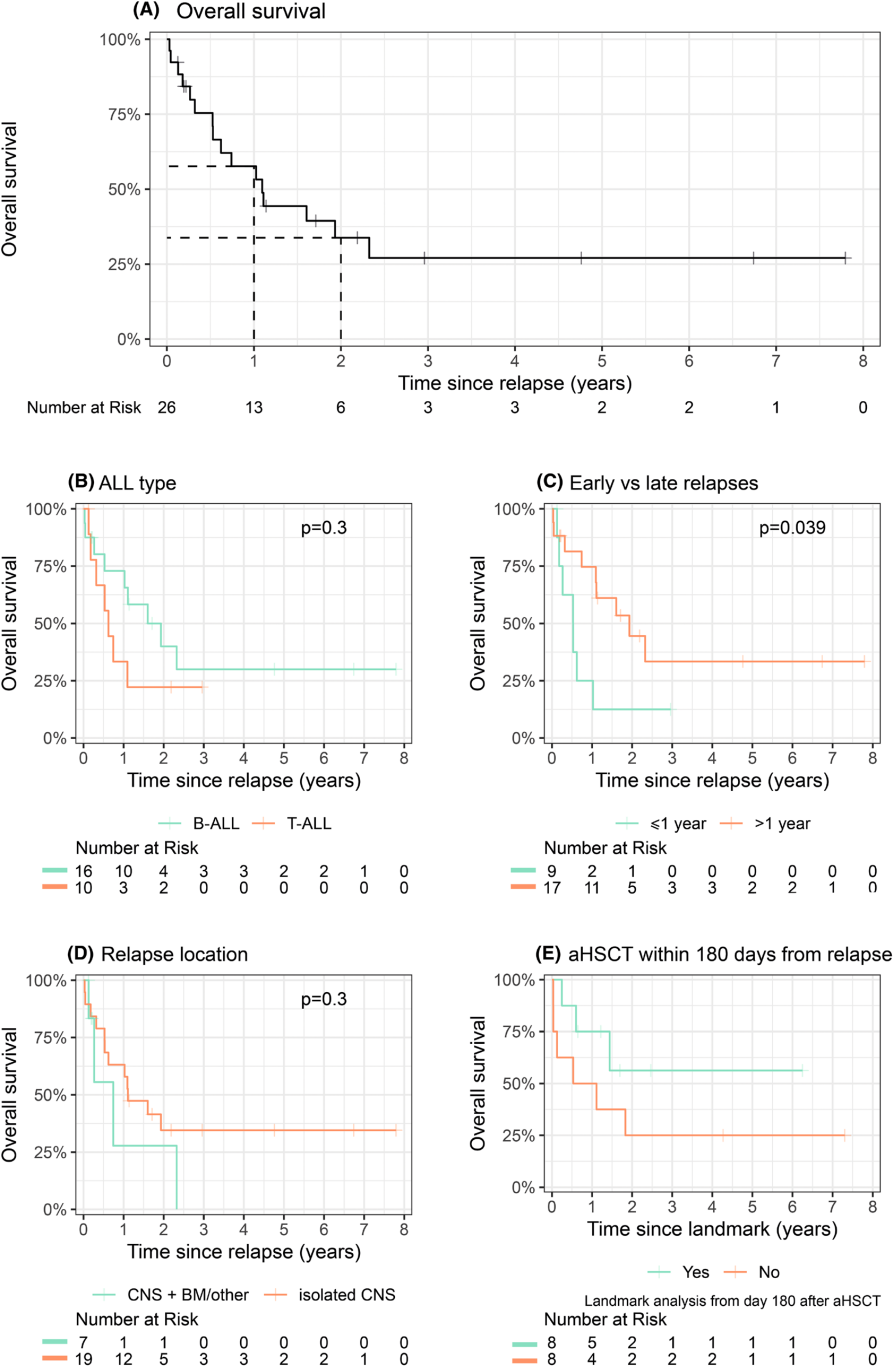
Survival after central nervous system (CNS) relapse. (A) Overall survival (OS) in all relapsed patients (1‐ and 5‐year OS estimates: 58% and 27%). (B–D) OS stratified by acute lymphoblastic leukaemia (ALL) type, early and late (≤1 vs. >1 year) CNS relapse and relapse location (CNS + bone marrow (BM)/other vs. isolated CNS). *p*‐values from log rank tests. (E) Landmark analysis at 180 days after allogeneic haematopoietic stem cell transplantation (aHSCT) comparing transplanted patients (*n* = 8) to those who were given reinduction treatment but were not transplanted (*n* = 8). Patients who died (*n* = 5) or were not censored (*n* = 3) before the landmark were included.

Median OS after CNS relapse was 13 months (95% CI 6.3 to not estimable), with a 1‐year OS of 58% (95% CI 41%–82%) and a 2‐year OS of 34% (95% CI 19%–61%, Figure 4A). The OS was comparable to patients without CNS involvement at first relapse (*n* = 135) in both univariable and multivariable analyses (Figure S4 and Table S3).

Survival after CNS relapse was similar in T‐ALL versus B‐ALL patients and isolated versus non‐isolated CNS relapses, but inferior in patients with a CNS relapse within 1 year after diagnosis (1‐year OS 25% vs. 75%, *p* = 0.039; Figure 4A–D). In a landmark analysis with follow‐up starting 180 days after relapse, we compared patients who underwent aHSCT before the landmark (*n* = 8) to those who were given reinduction treatment but were not transplanted (*n* = 8). After landmark, 2‐year OS was 56% in transplanted patients, compared to 25% in non‐transplanted patients (Figure 4E).

## DISCUSSION

To our knowledge, this is the first population‐based analysis of CNS involvement and relapse in adult ALL patients within a consecutive and completely unselected patient cohort. In view of the prospectively registered data, relatively large cohort, wide age span, chemotherapy‐based CNS prophylaxis and treatment according to modern and consistent national guidelines, we believe that the external validity of our results is high.

This study confirms previous reports demonstrating that high initial WBC count is an independent risk factor associated with both CNS involvement at diagnosis^7^, ^12^, ^14^ and CNS relapse.^5^, ^13^ In contrast, we found no such associations for either *BCR::ABL1*, previously associated with CNS involvement and relapse,^13^ or *KMT2A::AFF1*. This lack of association may be due to low power, or in the case of CNS relapse, a protective effect of frequent aHSCT in CR1 in both *BCR::ABL1*‐ and *KMT2A::AFF1*‐positive patients. However, it is unlikely that these results are caused by missing information in the register, as 92% of included patients underwent genetic testing and the frequencies of these aberrations in our material are similar to previous reports.^25^, ^26^ Several preclinical studies have demonstrated that CNS infiltration is not a feature of individual disease clones, but rather a generic property of ALL blasts.^27^, ^28^ The linear effect of WBC count on CNS involvement seen in our study supports these findings by suggesting that more proliferative leukaemias have a higher chance of being detected in the CNS simply because of increased risk that enough blasts have entered to surpass the limit of detection.

Neither chance of achieving CR, incidence of relapse nor overall survival was impacted by CNS involvement at diagnosis in our study, in contrast to previously reported clinical trial analyses,^12^, ^14^ suggesting that contemporary ALL treatments can overcome the adverse risk associated with CNS involvement without prophylactic CNS irradiation. In the Group for Research on Adult Acute Lymphoblastic Leukemia‐2005 trial,^14^ patients with CNS involvement had worse survival without increased relapse rates, suggesting excessive treatment‐related toxicity. In that trial, a larger proportion of patients with CNS involvement were consolidated with aHSCT in primary treatment, compared to our material, which may have contributed to the increased toxicity.

We report a 5‐year cumulative incidence of CNS relapse of 4.6% in adults with ALL, which is somewhat lower than previously reported^4^, ^5^, ^7^ despite including an unselected patient material with age up to 70 years. However, in contrast to previous reports, our analysis accounted for the competing risks of non‐CNS relapse and death, resulting in a lower but arguably more clinically relevant estimate.^29^ Interestingly, even though CNS involvement at diagnosis was more common in T‐ALL patients, stratified analysis demonstrated that it was only associated with an increased risk of CNS relapse in B‐ALL patients. While reduced power due to fewer patients with T‐ALL compared to B‐ALL could explain this result, it may suggest that contemporary treatment strategies for B‐ALL patients with CNS involvement lack some efficacy for preventing CNS relapse in these patients. This is an important finding in the context of the ongoing introduction of immunotherapies such as blinatumomab that seem to have low activity against CNS leukaemia in the upfront treatment of these patients.^30^ Replacing chemotherapy with immunotherapy is attractive in the perspective of treatment toxicity, but care must be taken to avoid increasing the risk of CNS relapse due to reduced prophylaxis. In addition, male sex was associated with a higher risk of CNS relapse. Male sex is a well‐known risk factor for poor outcomes in paediatric ALL^31^ and has previously been reported as a risk factor for CNS relapse in paediatric B‐ALL patients.^32^

While the survival of patients with CNS relapse in our population was somewhat better than previously reported (median OS about 13 months vs. 6 months),^3^, ^4^, ^7^ it remains poor, with only 34% 2‐year OS. In contrast to Fielding et al.,^3^ but not Reman et al.,^7^ the site of relapse did not impact overall survival, with comparable outcomes seen after non‐CNS relapse, isolated CNS relapse and combined CNS and bone marrow relapse. As demonstrated previously,^5^, ^33^ early CNS relapses are associated with worse outcomes. Over 50% of patients who underwent aHSCT within 180 days after CNS relapse survived beyond 2 years, supporting the use of transplantation as consolidation in CNS‐relapsed patients. We could not evaluate the efficacy of chimeric antigen receptor T‐cell (CAR‐T) therapy in this study since no CAR‐T‐cell products were nationally approved for treatment in first relapse during our study period.

This study has several strengths. First, it is comparatively large and population‐based, including consecutively registered, unselected ALL patients, which improves the generalizability of reported findings compared to clinical trial analyses. Second, the recently validated Swedish ALL register^21^ provides detailed clinical data with sufficiently long follow‐up time to detect relapses. Finally, vital status is provided by the Swedish population register, which is virtually complete with no loss to follow up and updated as of end of study. An important limitation is the absence of detailed information about primary treatment, which prevents associating CNS‐directed therapy with CNS relapse risk on the individual level. Furthermore, relapse treatments are absent from the register and therefore cannot be described. Finally, the register lacks information about whether evaluation for CNS involvement at relapse was conducted or not. Therefore, we cannot exclude that some patients that are labelled as having a relapse without CNS involvement may simply not have undergone CNS evaluation at relapse. This is somewhat mitigated by limiting the CNS relapse analysis to patients 70 years old or younger.

In conclusion, this study demonstrates that contemporary treatment without CNS irradiation can overcome most of the adverse prognosis associated with CNS involvement in patients with ALL. However, B‐ALL patients with CNS involvement still have an unmet need for preventing CNS relapse. In the future, highly sensitive diagnostics like CSF flow cytometry^34^ or cell‐free deoxyribonucleic acid analysis may improve residual CNS disease monitoring during therapy, but clinical risk factors such as male sex and WBC count at diagnosis can continue to play an important role in guiding early treatment decisions. To improve risk classification, future studies could aim to combine multiple such factors into a clinical risk score for CNS relapse.

## AUTHOR CONTRIBUTIONS

**Helene Hallböök:** Conceptualization; writing – review and editing; supervision; investigation. **Emma Bergfelt Lennmyr:** Conceptualization; writing – review and editing; investigation. **Erik Boberg:** Conceptualization; funding acquisition; writing – original draft; methodology; visualization; writing – review and editing; software; formal analysis; project administration; investigation. **Tove Wästerlid:** Conceptualization; writing – review and editing; supervision; methodology. **Anna Lübking:** Conceptualization; writing – review and editing; validation; data curation; investigation. **Karin E. Smedby:** Conceptualization; writing – review and editing; supervision; funding acquisition; methodology. **Joel Joelsson:** Conceptualization; writing – review and editing. **Caroline E. Dietrich:** Methodology; software; formal analysis; writing – review and editing.

## FUNDING STATEMENT

This work was supported by grants from Stockholm County Council (ALF, RS 2023‐0869) and Karolinska Institutet (FS‐2024:0017). Tove Wästerlid was supported by Region Stockholm (clinical researcher appointment). Emma Bergfelt Lennmyr was supported by Uppsala County Council (ALF).

## CONFLICT OF INTEREST STATEMENT

The authors have no conflicts of interest to disclose.

## Supporting information


**Figure S1.** Study flowchart. ALL, acute lymphoblastic leukaemia; B‐LBL, B‐lymphoblastic lymphoma; CNS, central nervous system; NOS, not otherwise specified.
**Figure S2.** Primary treatment by age, Philadelphia chromosome (Ph)‐status and year of diagnosis. Lighter areas with dotted borders denote patients that were labelled as ‘treated according to national guidelines’ in the register. The treatment protocols for these patients have been derived from these guidelines. In *BCR::ABL1*‐negative patients, the paediatric NOPHO ALL2008^1^ and ALLTogether1 protocols were most commonly used up to 45 years of age, while dose‐adjusted NOPHO ALL2008 was given to patients aged 46–65 years. *BCR::ABL1*‐positive patients aged up to 65 years were mainly treated with the ABCDV regimen^2^ combined with a tyrosine kinase inhibitor (mainly imatinib). Older patients were commonly treated with the EWALL‐inspired protocol^3^ or received palliative care. All remission‐inducing regimens included systemic high‐dose intravenous methotrexate (MTX) and/or cytarabine (ARA‐C), as well as intrathecal MTX (with or without addition of ARA‐C and prednisone) for CNS‐directed therapy. Cranial or craniospinal irradiation was not a part of primary treatment, except for high‐risk patients who were consolidated with allogeneic haematopoietic stem cell transplant (aHSCT) in first complete remission (CR1). Conditioning regimens generally included total body irradiation and usually without a cranial boost. The figure excludes patients where Ph status or primary treatment protocol is unknown (*n* = 87). EWALL: European Working Group for Adult ALL.
**Figure S3.** (A) Distribution of white blood cell (WBC) counts at diagnosis. (B) Cubic spline visualizing the association between WBC count at diagnosis and odds ratio (OR) for central nervous system (CNS) involvement, derived from simple logistic regression. (C) Cubic spline visualizing the association between WBC count at diagnosis and hazard ratio (HR) for CNS relapse, derived from Cox regression. At WBC counts >200, the relationships became non‐linear, but the uncertainty increased substantially due to very few patients having WBC counts in that range. In (B and C), data were therefore limited to WBC count <500. CNSi: central nervous system involvement, CNSr: central nervous system relapse.
**Figure S4.** Overall survival in relapsed patients, stratified by the presence of central nervous system (CNS) involvement at relapse. *p*‐value from the log rank test.
**Table S1.** Multivariable logistic regressions of associations between clinical factors and CNS involvement at diagnosis, stratified by ALL type.
**Table S2.** Cox regression for the association between CNS involvement and relapse and overall survival.
**Table S3.** Adjusted comparison of overall survival between patients with and without CNS involvement at relapse 1.

## Data Availability

Data supporting this study cannot be made available due to ethical restrictions.
