# Supplementary material for: Central nervous system involvement and relapse in adult acute lymphoblastic leukaemia—a population‐based analysis in 2007–2022
Source: Br J Haematol. 2026 Jun 1;209(2):589–600. doi: 10.1111/bjh.70589 (PMC13462185; doi:10.1111/bjh.70589)
Supplement: Supplementary file 1 [file BJH-209-589-s001.docx]

Supplementary material

## CNS involvement and relapse in acute lymphoblastic leukaemia - a population-based analysis 2007 - 2022

**Supplementary figure 1:** Study flowchart. ALL: Acute Lymphoblastic Leukaemia, NOS: Not Otherwise Specified, B-LBL: B-Lymphoblastic Lymphoma, CNS: Central Nervous System

**Supplementary figure 2:** Primary treatment by age, Philadelphia chromosome (Ph)-status and year of diagnosis. Lighter areas with dotted borders denote patients that were labelled as ”treated according to national guidelines” in the register. The treatment protocols for these patients have been derived from these guidelines. In *BCR::ABL1*-negative patients, the paediatric NOPHO ALL2008(1) and ALLTogether1 protocols were most commonly used up to 45 years of age, while dose-adjusted NOPHO ALL2008 was given to patients aged 46-65 years. *BCR::ABL1*-positive patients aged up to 65 years were mainly treated with the ABCDV regimen(2) combined with a tyrosine kinase inhibitor (mainly imatinib). Older patients were commonly treated with the EWALL inspired protocol(3) or received palliative care. All remission-inducing regimens included systemic high-dose intravenous methotrexate (MTX) and/or cytarabine (ARA-C), as well as intrathecal MTX (with or without addition of ARA-C and prednisone) for CNS-directed therapy. Cranial or craniospinal irradiation was not a part of primary treatment, except for high-risk patients who were consolidated with allogeneic haematopoietic stem cell transplant (aHSCT) in first complete remission (CR1). Conditioning regimens generally included total body irradiation and usually without a cranial boost. The figure excludes patients where Ph status or primary treatment protocol is unknown (n=87). EWALL: European Working Group for Adult ALL

**Supplementary figure 3:** A) Distribution of white blood cell (WBC) counts at diagnosis. B) Cubic spline visualizing the association between WBC count at diagnosis and odds ratio (OR) for central nervous system (CNS) involvement, derived from simple logistic regression. C) Cubic spline visualizing the association between WBC count at diagnosis and hazard ratio (HR) for CNS relapse, derived from Cox regression. At WBC counts >200, the relationships became non-linear, but the uncertainty increased substantially due to very few patients having WBC counts in that range. In B and C, data was therefore limited to WBC count < 500. CNSi: Central Nervous System involvement, CNSr: Central Nervous System relapse

**Supplementary figure 4:** Overall survival in relapsed patients, stratified by presence of Central Nervous System (CNS) involvement at relapse. P-value from the log rank test.

**Supplementary table 1:** Multivariable logistic regressions of associations between clinical factors and CNS involvement at diagnosis, stratified by ALL type.

| *Characteristic* | *B-ALL* | *T-ALL* |
| --- | --- | --- |
|  | **OR (95% CI)**^1^  (n=613) | **OR (95% CI)**^3^  (n=137) |
| **Age** |  |  |
| 17-45 | 1.00 (ref) | 1.00 (ref) |
| 46-60 | 1.18 (0.46-2.81) | 1.15 (0.24-4.40) |
| 61-70 | 1.76 (0.76-3.97) | **8.10 (2.14-31.8)** |
| 71+ | 0.98 (0.37-2.41) | 0.88 (0.04-5.58) |
| **Sex** |  |  |
| F | 1.00 (ref) | 1.00 (ref) |
| M | 1.56 (0.81-3.10) | 2.16 (0.66-8.10) |
|  | **OR (95% CI)**^2^  (n=587) | **OR (95% CI)**^4^  (n=125) |
| **WBC at diagnosis** |  |  |
| WBC<30 | 1.00 (ref) | 1.00 (ref) |
| WBC 30-100 | **2.35 (1.02-5.20)** | 2.76 (0.72-10.3) |
| WBC>100 | **3.04 (1.18-7.38)** | 1.67 (0.43-6.18) |
| ***KMT2A::AFF1* (B-ALL only)** | | |
| No | 1.00 (ref) |  |
| Yes | 1.06 (0.21-4.04) |  |
| ***BCR::ABL1* (B-ALL only)** | | |
| No | 1.00 (ref) |  |
| Yes | 0.90 (0.41-1.89) |  |
| **Bulky disease at diagnosis** |  |  |
| No |  | 1.00 (ref) |
| Yes |  | 0.62 (0.13-2.24) |

*^1^* B-ALL only. Estimated from a multivariable logistic regression model including age and sex

^2^ B-ALL only. Estimated from a multivariable logistic regression model including age, sex, WBC at diagnosis, *KMT2A::AFF1*, and *BCR::ABL1*

^3^ T-ALL only. Estimated from a multivariable logistic regression model including age and sex

^4^ T-ALL only. Estimated from a multivariable logistic regression model including age, sex, WBC at diagnosis, and bulky disease at diagnosis

^2,4^In the multivariable models 2 and 4, age was modelled as a cubic spline with 2 df.

ALL: Acute Lymphoblastic Leukaemia, OR: Odds Ratio, CI: Confidence Interval, WBC: White Blood Cell

**Supplementary table 2:** Cox regression for the association between CNS involvement and relapse and overall survival.

|  | *Relapse* | *Overall survival* |
| --- | --- | --- |
| **Characteristic** | **HR (95% CI)** *^1^*  (n=642) | **HR (95% CI)** *^1^*  (n=642) |
| **CNS involvement at diagnosis** |  |  |
| CNS- | 1.00 (ref) | 1.00 (ref) |
| CNS+ | 1.14 (0.67-1.93) | 0.97 (0.62-1.52) |

*^1^*Cox regression adjusted for: ALL type (T-ALL or B-ALL), t(4;11), WBC at diagnosis (<30, 30-100, >100), Age at diagnosis (cubic spline with 2 df), and Sex (F or M). HR: Hazard Ratio, CI: Confidence Interval, CNS: Central Nervous System

**Supplementary table 3:** Adjusted comparison of overall survival between patients with and without CNS involvement at relapse 1.

| Characteristic | HR (95% CI)^1^  (n=161) |
| --- | --- |
| CNS involvement at relapse 1 | |
| CNS | 1.0 (ref) |
| Non-CNS | 1.38 (0.79-2.41) |

^1^Cox regression adjusted for: ALL type (T-ALL or B-ALL), WBC at diagnosis (<30, 30-100, >100), Age at relapse (cubic spline with 2 df) and Sex (F or M). CI: Confidence Interval, HR: Hazard Ratio

**References**

1.	Toft N, Birgens H, Abrahamsson J, Griškevičius L, Hallböök H, Heyman M, et al. Results of NOPHO ALL2008 treatment for patients aged 1–45 years with acute lymphoblastic leukemia. Leukemia. 2018 Mar;32(3):3. doi:10.1038/leu.2017.265

2.	Hallböök H, Simonsson B, Ahlgren T, Björkholm M, Carneskog J, Grimfors G, et al. High-dose cytarabine in upfront therapy for adult patients with acute lymphoblastic leukaemia. Br J Haematol. 2002;118(3):748–54. doi:10.1046/j.1365-2141.2002.03685.x

3.	Rousselot P, Coudé MM, Gokbuget N, Gambacorti Passerini C, Hayette S, Cayuela JM, et al. Dasatinib and low-intensity chemotherapy in elderly patients with Philadelphia chromosome–positive ALL. Blood. 2016 Aug 11;128(6):774–82. doi:10.1182/blood-2016-02-700153
